# Agonist Antibody Converts Stem Cells into Migrating Brown Adipocyte-Like Cells in Heart

**DOI:** 10.3390/cells9010256

**Published:** 2020-01-20

**Authors:** Kyung Ho Han, Britni M. Arlian, Chih-Wei Lin, Hyun Yong Jin, Geun-Hyung Kang, Sahmin Lee, Peter Chang-Whan Lee, Richard A. Lerner

**Affiliations:** 1 Department of Chemistry, The Scripps Research Institute, La Jolla, CA 92037, USA; kyungho1.han@gmail.com (K.H.H.); cwlin@scripps.edu (C.-W.L.); 2 Department of Biomedical Sciences, University of Ulsan College of Medicine, ASAN Medical Center, Seoul 05505, Korea; 3 Departments of Molecular Medicine, Immunology and Microbiology, The Scripps Research Institute, La Jolla, CA 92037, USA; britni@scripps.edu; 4 Department of Urology, University of California, San Francisco, CA 94158, USA; jinhyunyong@gmail.com; 5 Division of Cardiology, Asan Medical Center Heart Institute, University of Ulsan College of Medicine, Seoul 05505, Korea; rkdrmsgud@nate.com (G.-H.K.); sahmin.lee@amc.seoul.kr (S.L.)

**Keywords:** antibody, cell differentiation, Iodotyrosine Deiodinase (IYD), brown adipocyte-like cells

## Abstract

We present data showing that Iodotyrosine Deiodinase (IYD) is a dual-function enzyme acting as a catalyst in metabolism and a receptor for cooperative stem cell differentiation. IYD is present both in thyroid cells where it is critical for scavenging iodine from halogenated by-products of thyroid hormone production and on hematopoietic stem cells. To close the cooperative loop, the mono- and di-Iodotyrosine (MIT and DIT) substrates of IYD in the thyroid are also agonists for IYD now acting as a receptor on bone marrow stem cells. While studying intracellular combinatorial antibody libraries, we discovered an agonist antibody, H3 Ab, of which the target is the enzyme IYD. When agonized by H3 Ab, IYD expressed on stem cells induces differentiation of the cells into brown adipocyte-like cells, which selectively migrate to mouse heart tissue. H3 Ab also binds to IYD expressed on human myocardium. Thus, one has a single enzyme acting in different ways on different cells for the cooperative purpose of enhancing thermogenesis or of regenerating damaged heart tissue.

## 1. Introduction

An organism is an amalgamation of different cell types that collectively regulate all of its functions. Each cell type operates by using a different set of molecules of which the output becomes part of the collective function of the organism. Thus, each cell has a special task of which the nature is determined by its differentiated state. However, another level of efficiency could be achieved if proteins could have multiple functions that were involved synergistically in both the chemistry and biology of a physiological task. For instance, an enzyme could be used for chemical transformations and could serve as a receptor for the differentiation of new cells with synergistic capabilities.

Recently, we discovered such a dual-function enzyme while studying intracellular combinatorial antibody libraries that regulate cell fates, particularly when autocrine selections are used [1,2,3,4,5,6]. We have previously applied this system to isolate antibodies capable of differentiating stem cells into specific immune cell types, of inserting functional proteins within proteins, and of serving as a fluorescent cell reporter to identify ligand and receptor interactions [1,2,3,4,5,6]. Here, we wanted to select antibodies that regulate migration of hematopoietic stem cells to peripheral tissues. Such an agonist antibody was found of which the target is Iodotyrosine Deiodinase (IYD).

IYD is expressed on thyroid cells where it plays a critical role in thyroid hormone production as well as on hematopoietic stem cells. The interesting nuance is that the physiological substrate of the enzyme is also a ligand that fosters differentiation of human and murine stem cells into brown adipocyte-like cells that migrate to the heart. Either the agonist antibody, H3 Ab, or the substrate of IYD can induce differentiation. Since the antibody alone acting as an orthogonal agonist can induce differentiation, IYD appears to be a true receptor.

The induction of differentiation and regulation of metabolism appear to be independent functions of IYD. Nevertheless, the physiological consequences of the enzyme acting as a classical catalyst or as a receptor are synergistic because the chemistry catalyzed by the enzyme or its role as a receptor inure to the generation of molecules and new cells that have the same physiological mission. Thus, our results suggest that both the thyroid metabolites and newly differentiated bone marrow stem cells play a crucial role in thermogenesis or damaged heart tissue regeneration.

## 2. Materials and Methods

### 2.1. Mouse Strains and Cell Lines

These mouse strains were purchased from The Jackson Laboratory (Sacramento, CA, USA): C57BL/6J, FVB/NJ, B6 (Cg)-*Tyr^c-2J^* Tg (UBC-mCherry) 1Phbs/J, FVB-Tg (CAG-luc,-GFP) L2G85Chco/J, and CByJ.B6-Tg (UBC-GFP) 30Scha/J. HEK293T and TC1 cells were cultured in DMEM (Invitrogen, Carlsbad, CA, USA) supplemented with 10% Fetal Calf Serum (HyClone, Chicago, IL, USA) and 1% penicillin and streptomycin (Invitrogen, Carlsbad, CA, USA). Expi293F cells were cultured in Expi293 Expression Media (Invitrogen, Carlsbad, CA, USA). Human CD34^+^ cells (AllCells, Alameda, CA, USA) were purchased and reported to be more than 96% pure. Murine bone marrow cells were maintained in StemSpan SFEM supplemented with CC100 (STEMCELL Technologies, Vancouver, BC, Canada), SFEM without supplement, or RPMI (Invitrogen, Carlsbad, CA, USA) with 1% Fetal Calf Serum (FCS). Animal protocols were approved by the Institutional Animal Care and Use Committee of The Scripps Research Institute (12-0029) or by the Institutional Ethics Committee and Institutional Animal Care Committee of the University of Ulsan College of Medicine (2016-02-168, 2017-12-281).

### 2.2. Human Heart Tissues

Informed consent was received from patients, and protocols were approved by the Institutional Review Board of Asan Medical Center and the University of Ulsan College of Medicine (2017-0556) prior to use of human heart tissues for experiments.

### 2.3. Combinatorial Antibody Library

Single-chain variable fragment (ScFv) genes from a naïve human combinatorial antibody library (1 × 10^11^ genes) were sub-cloned into the pLV2 lentiviral vector. HEK293T cells were then co-transfected with the lentiviral vectors pCMVD8.91 and pVSVg to produce lentiviral antibody.

### 2.4. Bone Marrow Transduction and Transplantation

Murine bone marrow cells were infected for 3 days at 37 °C with our lentiviral antibody library at a multiplicity of infection (MOI) = 2. The transduced cells were then transplanted to lethally irradiated mice. After 2–3 weeks, the mice were perfused with Phosphate-buffered saline (PBS) and fixed with 2% paraformaldehyde (Sigma, St. Louis, MO, USA). The hearts were harvested and stored at −80 °C until homogenates were analyzed by PCR using primers specific for the vector. Amplified PCR products were then visualized by gel electrophoresis and extracted for further analysis.

### 2.5. Purification of Single-Chain Variable Fragment—Fc Proteins

Expi293F cells (Invitrogen, Carlsbad, CA, USA) were transfected with the H3 Ab-Fc tag fusion protein for transient gene expression. H3 antibodies were purified by protein G affinity chromatography (ÄKTAxpress system) with the HiTrap Protein G HP column (GE Healthcare, Chicago, IL, USA), dialyzed in PBS (pH 7.4), and stored at 4 °C.

### 2.6. Immunoprecipitation and Mass Spectrometry

Murine bone marrow cells were harvested and solubilized in lysis buffer prior to incubation with H3 Ab for 2–4 h in a cold room. Lysates were then incubated with 50 μL of Protein G Sepharose beads (Pierce, Rockford, IL, USA) and eluted into a linear trap quadrupole mass spectrometer (Thermo Scientific, Waltham, MA, USA) with a 2-kV electrospray voltage source. From a full MS scan (400–2000 *m*/*z*), the three most intense ions were then chosen for additional MS/MS analysis. Raw data from the full MS scan were analyzed using the MASCOT (http://www.matrixscience.com/) search engine.

### 2.7. Western Blot

Bone marrow and CD34^+^ cells were lysed in buffer (50 mM Hepes, pH 7.2, 150 mM NaCl, 50 mM NaF, 1 mM Na_3_VO_4_, 10% glycerol, and 1% Triton X-100) after being washed in cold PBS. Lysates were then clarified prior to quantification of proteins using the BCA assay (Pierce, Rockford, IL, USA). Proteins were then separated by SDS/PAGE and transferred to a membrane using the iBlot blotting system (Invitrogen, Carlsbad, CA, USA). IYD protein (Fitzgerald, North Action, MA, USA), IYD DNA (OriGene, Rockville, MD, USA), IYD shRNA (OriGene, Rockville, MD, USA), β actin, phospho-AKT, ERK, and p38 (Cell Signaling Technology, Danvers, MA, USA) were used for Western blotting as described previously [1].

### 2.8. Flow Cytometry

Bone marrow and CD34^+^ cells were stained with anti-GFP and anti-mCherry (Invitrogen) and analyzed by flow cytometry (LSRII, Becton Dickinson, Franklin Lakes, NJ, USA).

### 2.9. Real Time Quantitative (RT-q) PCR

Total RNA was extracted (Qiagen, Germantown, MD, USA) from cells that had been cultured with H3 Ab to synthesize cDNA (Bio-Rad Laboratories, Hercules, CA, USA). Samples (in triplicate) containing 400 ng of cDNA were amplified in SYBR Green Supermix using a C1000 Thermal cycler (Bio-Rad Laboratories). Primers were designed to identify human UCP1, PGC1A, PRDM16, AP2 (FABP4), adipoQ, and PPARG. Primer sequences are provided in Supplementary Table S2.

### 2.10. Microscopy

Frozen whole heart tissues were cut horizontally, and sections were incubated on coverslips overnight with rat anti-mCherry (1:500, Invitrogen, Carlsbad, CA, USA), rabbit anti-UCP1 (1:500, Invitrogen, Carlsbad, CA, USA), or rabbit anti-IYD (1:500, Proteintech, Rosemont, IL, USA). Washed sections were then incubated with secondary antibody (goat anti-rabbit or anti-rat, 1:250, Invitrogen, Carlsbad, CA, USA) for 1 h. Washed coverslips were mounted onto slides using medium containing DAPI (Invitrogen, Carlsbad, CA, USA) and imaged with a Zeiss LSM 710 laser scanning confocal microscope. Immunofluorescence was performed on cultured cells with Lipidtox (Thermo Fisher, Waltham, MA, USA). Microscopy was performed using an IN Cell Analyzer 6000 (GE Healthcare, Chicago, IL, USA).

### 2.11. Bioluminescence Imaging

Bone marrow cells were harvested from luciferase-expressing transgenic mice (FVB-Tg (CAG-luc,-GFP) L2G85Chco/J) and infected with the H3 Ab lentiviral vector prior to transplantation into fatally irradiated recipient mice (FVB/NJ). After 1 week, the recipient mice were injected i.v. with CycLuc1 (100 μL of 5 mM solution in PBS; END Millipore, St. Louis, MI, USA) and imaged with the IVIS Lumina^®^ system (Perkin-Elmer, Waltham, MA, USA).

### 2.12. Electron Microscopy

Heart tissues were fixed in glutaraldehyde (2.5%) cacodylate (0.1 M) buffer and then rinsed, filtered, and mounted on scanning electron microscope slides coated with iridium. Analysis was performed using a Hitachi S-4800 scanning electron microscope (Hitachi High Technologies, Clarksburg, MD, USA).

### 2.13. RNA Sequencing and Data Analysis

Total RNAs were isolated in replicates of three from human CD34^+^ cells mixed with the control antibody, of five from hCD34^+^ cells incubated with H3 Ab, and of three from hCD34^+^ cells incubated with M-CSF. Following the manufacturer’s protocol, RNA-Seq libraries were prepared using the NEBNext^®^ Ultra™ Directional RNA Library Prep Kit (Illumina, San Diego, CA, USA). Briefly, for each sample, 500 ng total RNA was polyA selected and converted to double-stranded cDNA, followed by fragmentation and ligation of sequencing adapters. The library was then PCR amplified with barcoded PCR primers, purified, and size selected with AMPure XP Beads prior to Illumina NextSeq500 analysis (Illumina, San Diego, CA, USA). The expression levels of human transcripts were estimated using Salmon (BioRxiv, Laurel Hollow, NY, USA). Statistical analyses were done with edgeR (Bioconductor, open source software), and the differential expression genes were classified with false-discovery rates < 0.05, absolute fold change > 2, and averaged CPM (counts per million) > 1 in the samples. The heatmap was built using Cluster3 (1.59-1, Ubuntu, open source software) and JavaTreeView (3.0, Sourceforge, open source software).

## 3. Results

### 3.1. In Vivo Selection of Antibodies that Induce Cell Migration

**Figure 1:**
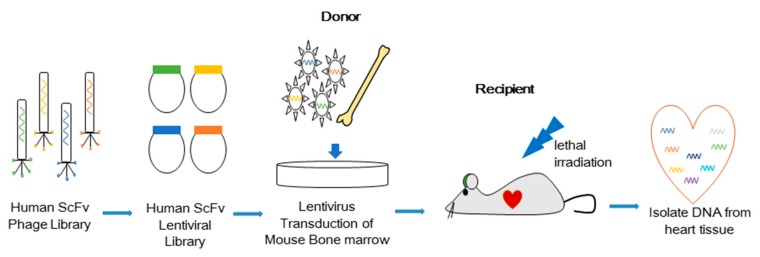
Scheme for in vivo selection of the H3 antibody that regulates migration of cells to the mouse heart: Single-chain variable fragment (ScFv) genes from a human phage library (10^8^ members) were transferred into the pLV2 lentiviral vector, which attaches antibody molecules to the cell surface. Since each cell displays a different antibody on its surface with the potential to have a unique target, this selection system is autocrine based. The lentiviral antibody library was used to transduce total murine bone marrow cells in vitro prior to injecting them into fatally irradiated wildtype C57BL/6J mice. After 7 days, mouse hearts were collected and analyzed by PCR to find antibody genes in differentiated cells that migrated to the heart.

We have devised a method to isolate antibodies that control cell fates using intracellular combinatorial libraries in vitro [2,6,7]. Previously, a novel in vivo selection method was used to identify antibodies that induce hematopoietic stem cells to both differentiate into microglia and to selectively migrate to the brain [1]. Here, we studied (Figure 1) an unbiased in vivo selection to identify an agonist antibody that induces bone marrow cells to traffic to the heart. Briefly, an unbiased antibody library (cell surface plasma membrane binding format) in lentiviruses containing approximately 10^8^ diverse members was used to transduce mouse bone marrow cells [6]. The transduced cells were then injected into fatally irradiated mice to observe cell migration to the heart. After 7 days, the mice were perfused with PBS and their hearts were collected to prepare genomic DNA. The human ScFv sequences integrated into the cells were recovered by PCR. The H3 antibody (H3 Ab) gene was isolated for further study, since it was seen five times in cells that trafficked to the heart. The H3 antibody gene sequence was not found in the other organs analyzed (Figure S1).

### 3.2. Isolated Antibody H3 Induces Migration of Cells to the Heart

**Figure 2:**
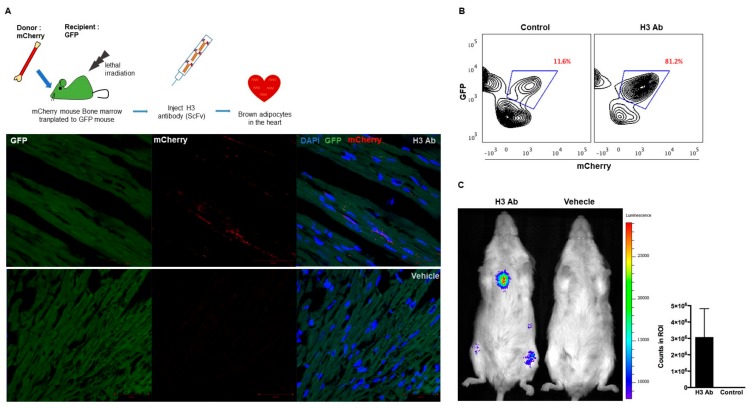
H3 antibody induces cell migration: (**A**) Research design used to investigate the migration of differentiated HSCs from mouse bone marrow to heart. Bone marrow from mCherry^+^ mice was adoptively transplanted into lethally irradiated GFP^+^ mice, which were then injected with H3 Ab (50 μg/mouse, i.p., two times/week) for 3 weeks. Following the treatment, hearts were harvested from perfused mice and analyzed by immunofluorescence histochemistry. GFP^+^ heart tissue sections (10 μm) were incubated with anti-mCherry and DAPI antibodies and then scanned by confocal microscopy. Remarkably, more mCherry^+^ cells were found in the hearts of mice treated with H3 Ab versus vehicle. Scale bars = 50 μm. (**B**) Harvested hearts were analyzed by flow cytometry. Analysis showed that more donor mCherry^+^ cells migrated to the heart in the GFP^+^ mice injected with H3 Ab versus vehicle alone. (**C**) Representative images (1 week posttransplantation) of FVB/NJ mice transplanted with luc^+^ bone marrow cells that were infected in vitro with the H3 Ab lentiviral vector or no Ab (control). Regions of Interest (ROIs) were used to quantify counts (mean ± s.d.; *n* = 5).

To show that binding of H3 Ab to mouse hematopoietic stem cells causes them to traffic to the heart, H3 Ab was expressed from a mammalian vector and purified. Donor mCherry^+^ mouse bone marrow cells were harvested and injected into irradiated GFP^+^ mice. Then, purified H3 Ab (50 μg/mouse, intraperitoneal injection (i.p.), two times/week) was injected into transplanted GFP^+^ mice for 3 weeks. The mice were then perfused to look for cells in the heart ubiquitously expressing mCherry (Figure 2A and Figure S2A). Harvested hearts were cut into 2 hemispheres for analysis by flow cytometry and immunohistochemistry (IHC) staining. Analyses showed donor mCherry^+^ cells from mice injected with the H3 Ab migrated to the heart (Figure 2B). Similarly, mCherry^+^ total bone marrow cells that were transduced with an H3 Ab lentiviral vector in vitro and then adoptively transferred into fatally irradiated GFP^+^ mice also migrated to the heart (data not shown).

To confirm that the integrated H3 Ab gene induced the mouse hematopoietic stem cells to traffic to the heart, we transduced bone marrow cells from luciferase-expressing (luc^+^) mice with H3 Ab lentivirus, injected them into fatally irradiated FVB/NJ mice, and looked for luc^+^ cells after 1 week by bioluminescent in vivo imaging. Not surprisingly, we found that donor luc^+^ cells transduced with the H3 Ab trafficked to the heart (Figure 2C and Figure S2B).

### 3.3. Purified H3 Antibody Transforms Human Hematopoietic Stem Cells into Brown Adipocyte-Like Cells

**Figure 3:**
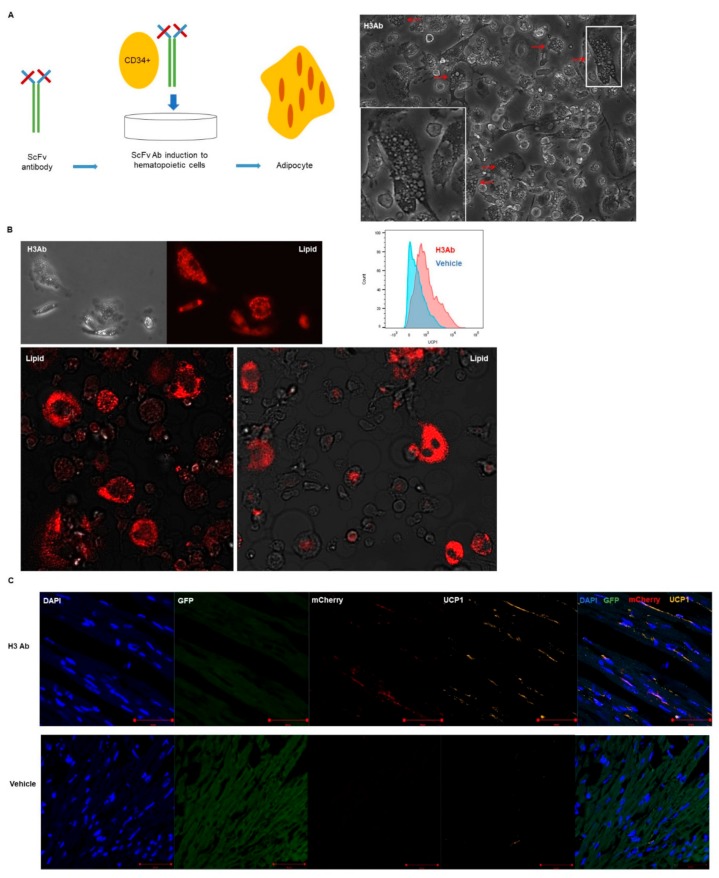

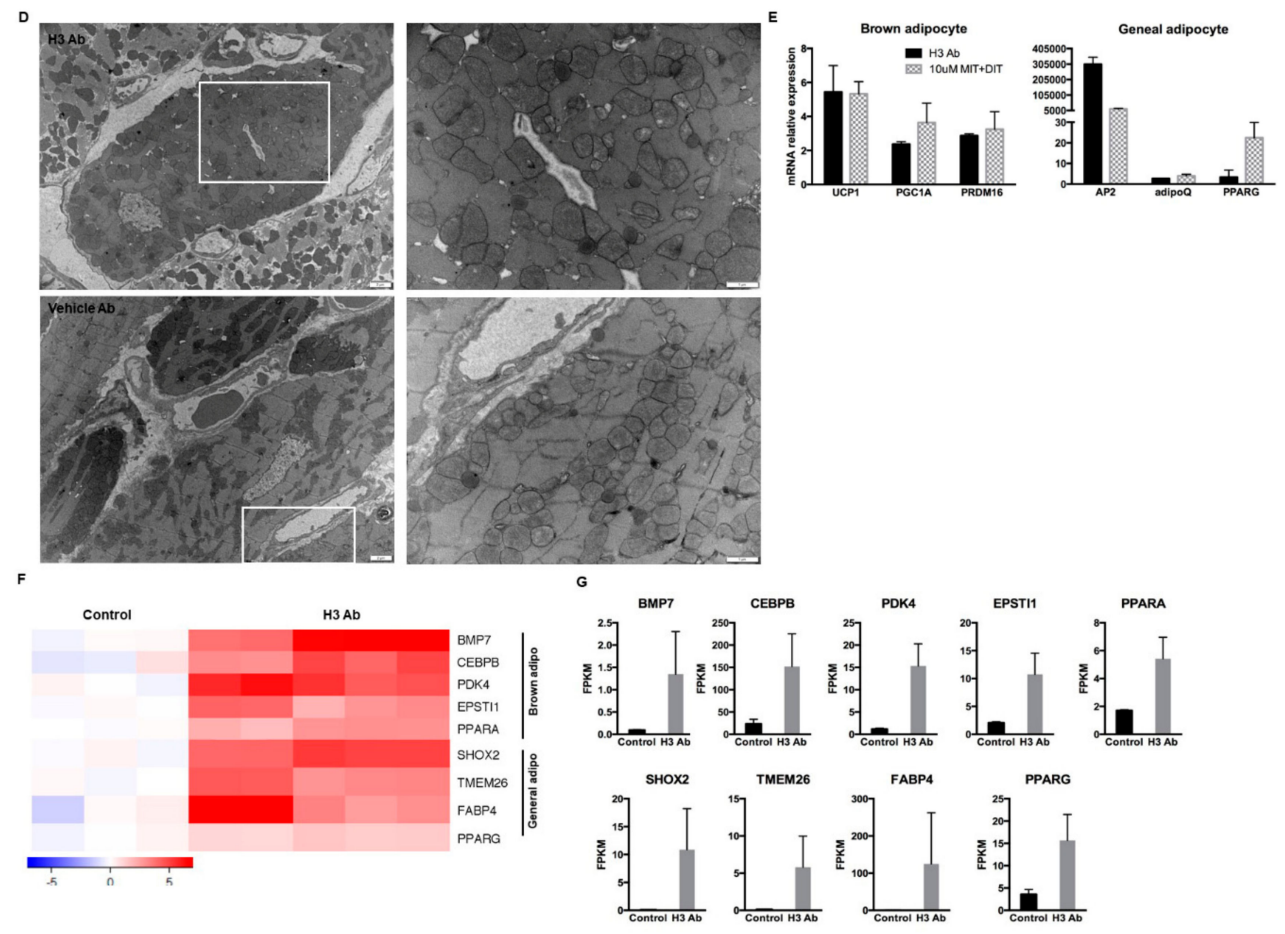
H3 antibody differentiates human CD34^+^ cells into brown adipocyte-like cells. (**A**) Human CD34^+^ hematopoietic stem cells that were treated with H3 antibody for 2 weeks in vitro differentiated into brown adipocyte-like cells. The white box indicates the region magnified in the insert. Red arrows point to adipocyte-like cells (Magnification: 20×). (**B**) Human CD34^+^ cells treated in vitro for 2 weeks with purified H3 Ab stained positive for large lipid droplets (Magnification: 20×) and expressed brown adipocyte marker UCP1 as confirmed by flow cytometry. (**C**) To show migration of the adipocyte-like cells from the bone marrow to the heart, H3 Ab (50 μg/mouse, i.p., two times/week) was injected into transplanted GFP^+^ mice for 3 weeks. After perfusion. hearts were harvested and frozen in OCT blocks for immunofluorescence histochemistry. Heart tissue sections (10 μm) were incubated with anti-UCP1 and DAPI and then scanned by confocal microscopy. As expected, UCP1 staining was notably stronger in heart tissue from mice injected with H3 Ab compared to untreated controls. Scale bars = 50 μm. (**D**) Numerous mitochondria, another characteristic of brown adipocytes, were also detected by electron microscopy in the differentiated cells from hearts of C57BL/6J mice treated for 3 weeks with H3 Ab (50 μg/mouse, i.p., two times/week). The white boxes indicate the regions magnified in the images to the right. Scale bars = 1 or 2 μm. (**E**) Total RNA was extracted from human CD34^+^ cells that had been incubated for 2 weeks with H3 Ab or Iodotyrosine Deiodinase (IYD) substrates to perform qRT-PCR. The analysis showed that the treated cells had a characteristic adipocyte mRNA expression profile with comparable relative mRNA levels of brown adipocyte (UCP1, PGC1A, and PRDM16) and general adipocyte (AP2, adipoQ, and PPARG) gene markers. (**F**) Brown and general adipocyte gene expression as determined by RNA sequencing analysis: The hierarchal clustering heat map demonstrates a unique expression pattern in the H3 Ab-treated cells when compared to isotype Ab (control)-treated cells. The expression profile of the induced brown adipocytes is consistent with previous reports. (**G**) Highly expressed brown adipocyte markers from the RNAseq analysis (*n* = 3 and 5) are summarized as Fragments Per Kilobase of transcript per Million mapped reads (FPKM).

To test whether purified H3 antibody could alone differentiate human stem cells, human CD34^+^ cells were mixed with H3 Ab for two weeks in vitro. In the presence of purified H3 antibody, human CD34^+^ cells were transformed into cells that resemble adipocytes, that express the brown adipocyte marker uncoupling protein 1 (UCP1) (Figure 3A), and that stain positive with lipid droplet staining similar to adipocytes (Figure 3B).

To further study the stem cells that migrated to the heart, heart sections were stained with DAPI and anti-UCP. The number of mCherry^+^ UCP1^+^ cells that migrated to the heart of irradiated GFP^+^ mice treated with H3 Ab was significantly higher than in control animals treated with vehicle alone (Figure 3C and Figure S3A). In addition, we scanned the heart sections by electron microscopy to address the question of mitochondrial number and found that the mice treated with H3 Ab had more mitochondria than controls (Figure 3D and Figure S4).

We obtained mRNA sequences from human CD34^+^ cells treated in vitro with purified H3 Ab or left untreated and compared those mRNA profiles to published gene expression data from previous studies on brown adipocytes [8,9,10]. The high selectivity of our H3 antibody for differentiation of adipocytes was verified by qRT-PCR by comparing its profile to cells that were differentiated in vitro by addition of IYD substrate instead of H3 antibody. The analysis showed that the cells induced by H3 antibody or IYD substrate expressed mRNA for the brown adipocyte markers UCP1, PGC1A, and PRDM16 and the general adipocyte markers AP2, adipoQ, and PPARG (Figure 3E). Notably, we found genes in our induced cells that are highly expressed in brown adipocytes, including PDK4, EPSTI1, ACVR2B, ADRBK1, ARDC3, ATG7, ATF4, BACE1, EIF4EBP2, FOXO1, ID1, LRP6, and NR1H3. These findings are consistent with previous reports (Figure 3F,G; Figure S3B,C; and Table S1).

### 3.4. H3 Ab has a Novel Target

**Figure 4:**
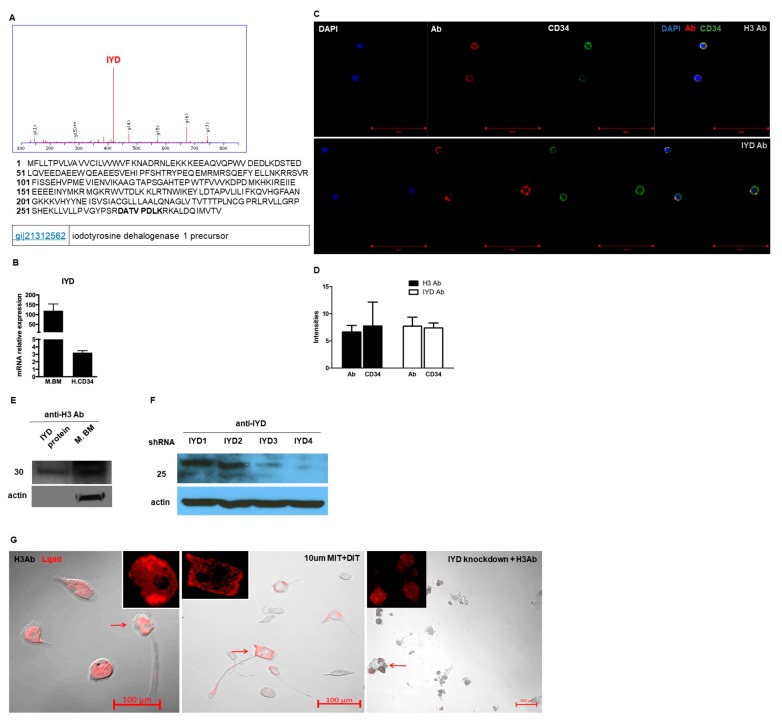
IYD is the novel target of the H3 Ab. (**A**) Murine bone marrow lysates were mixed with H3 Ab to immune-precipitate binding proteins and were then analyzed by silver staining and Nano-LC-MS/MS. IYD peptides are highlighted in bold. (**B**) Total RNA was extracted from murine bone marrow and human CD34^+^ cells to compare IYD expression by qRT-PCR. (**C**) Human CD34^+^ cells were stained with DAPI, H3 Ab, commercial anti-IYD, or anti-CD34 antibody to confirm surface expression of IYD by confocal microscopy. Scale bars = 50 μm. (**D**) Graph of fluorescence intensities from staining hCD34^+^ cells with H3 Ab or commercial IYD Ab. (**E**) The H3 Ab recognized human IYD protein and murine bone marrow lysates when analyzed by Western blotting. (**F**) IYD commercial antibody bound to IYD shRNA-treated cell lysates in Western blots. (**G**) Mouse bone marrow cells were incubated with H3 Ab, IYD substrates, or H3 Ab + IYD shRNA. Staining with the Lipidtox adipocyte marker revealed no cell differentiation in H3 Ab + IYD shRNA-treated bone marrow. Red arrows indicate adipocyte-like cells (Magnification: 20×).

To determine the target molecule recognized by the H3 antibody, H3 antibodies were purified using a mammalian Expi293F expression system. Purified H3 antibody was mixed with mouse bone marrow lysates, and immune complexes were precipitated on a protein G column. Proteins that reacted with the H3 antibody were analyzed by mass spectrometry (MS). Six candidate hits were found (Figure 4A). Iodotyrosine dehalogenase 1 precursor (IYD) was one of the top hits and is a membrane binding protein [11]. IYD expression on mouse bone marrow and human CD34^+^ cells were confirmed by qRT-PCR analysis (Figure 4B). Further, IYD expression on human CD34^+^ cells were confirmed by staining them with H3 Ab or a commercially available antibody against IYD and by imaging them with a confocal microscope (Figure 4C,D). To verify that IYD was the correct target antigen of H3 Ab, purified human IYD protein as well as IYD expressed on mouse bone marrow cells were detected with H3 Ab by Western blotting (Figure 4E). To further verify the target, we stably knocked down IYD expression using shRNA in TC1 cells (Figure 4F). Wild-type bone marrow was incubated for 2 weeks with H3 Ab alone, IYD substrates MIT/DIT, or H3 Ab with IYD shRNA. By lipid staining, we confirmed that incubation with either H3 Ab alone or IYD substrates induced differentiation of mouse bone marrow into brown adipocyte-like cells; however, there was no formation of adipocyte-like cells when the cells were co-incubated with H3 Ab and IYD shRNA (Figure 4G).

### 3.5. H3 Ab Detects IYD in Human Heart

**Figure 5:**
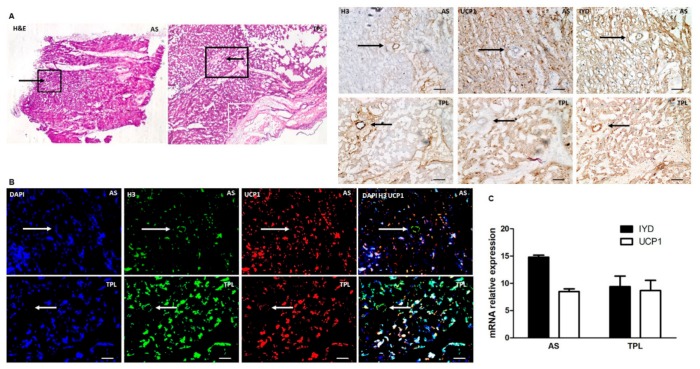
IYD detected by H3 Ab in human heart: (**A**) Human myocardial heart tissues from Aortic stenosis (AS) and Transplantation (TPL) patients were stained with H&E or H3 Ab, UCP1, and IYD antibodies for immunohistochemical analysis. The black boxes indicate the magnified regions analyzed in subsequent immunohistochemistry (IHC) images. (**B**) The same tissues were prepared for immunofluorescence analysis by confocal microscopy using the H3 Ab, UCP1 Ab, commercially available IYD Ab, and DAPI. Black and white arrows indicate human myocardium blood vessels. Scale bars = 100 μm. (**C**) Total RNA was isolated from heart tissues of AS and TPL patients to confirm expression of IYD and UCP1 by qt-PCR analysis.

Since we showed that injected cells migrated to the heart and are UCP1 positive in the mouse model, we next investigated whether IYD and UCP1 could be detected in human myocardial tissues by immunohistochemistry (Figure 5A), immunofluorescence (Figure 5B), and qt-PCR analysis (Figure 5C). All analyses confirmed that both IYD and UCP1 are notably expressed in human heart tissue. Further, the target antigen IYD was detected by the H3 Ab in human myocardium.

### 3.6. IYD Substrates MIT and DIT Differentiate HSCs into Brown Adipocyte-Like Cells

**Figure 6:**
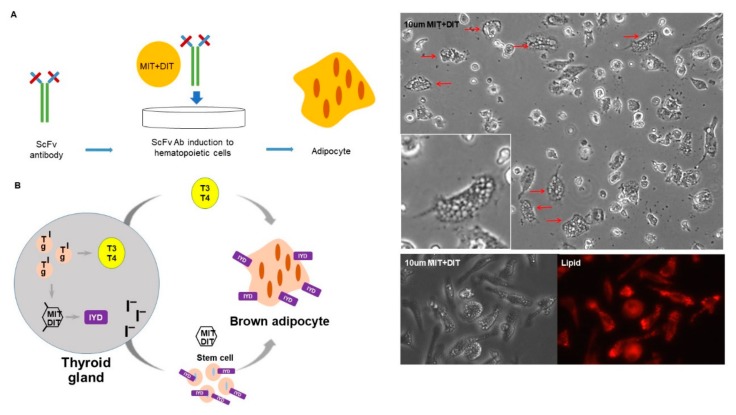
MIT and DIT induced stem cell differentiation into brown adipocyte-like cells. (**A**) The hematopoietic stem cell differentiation by IYD substrate (MIT + DIT): In the presence of IYD substrate for 2 weeks, human CD34^+^ cells differentiated into adipocyte-like cells as measured by lipid staining. Red arrows indicate adipocyte-like cells. (Magnification: 20×). (**B**) Proposed scheme for the dual role of IYD as a catalyst in thyroid cells and as a receptor for cooperative stem cell differentiation: IYD scavenges iodine and, in the presence of the MIT/DIT substrate, catalyzes the production of Triiodothyronine (T3) and Thyroxine (T4) thyroid hormones. T3 and T4 signaling can then lead to increased brown adipogenesis. IYD-expressing stem cells function as a receptor for H3 Ab or MIT/DIT to bind and induce differentiation of the stem cell into brown adipocytes.

IYD plays a major role in scavenging iodine, which is essential for thyroid hormone homeostasis in the human body. 3-monoiodo-l-tyrosine (MIT) and 3,5-diodo-l-tyrosine (DIT) are substrates of thyroid hormone production and catalyzed by IYD [12,13]. No additional roles for MIT/DIT have been reported, but we hypothesized that adding these substrates directly to human CD34^+^ cells should induce differentiation of the cells into adipocyte-like cells similar to when the cells are incubated with H3 Ab. To this end, human CD34^+^ cells were mixed with the IYD substrates MIT and DIT for two weeks in vitro. The substrates did, in fact, transform the human CD34^+^ cells into cells with the morphology of adipocytes (Figure 6A).

A major thesis of this report is that the metabolic enzyme, IYD, in thyroid cells also serves as a receptor for stem cell differentiation. In order to be part of a physiological circuit, this enzyme receptor when activated should cause differentiation into cells with functions synergistic to those of the thyroid. Further, to link the pathways, the metabolic enzyme should use the same substrates it uses in the thyroid, albeit for a different purpose (Figure 6B).

## 4. Discussion

Generally, we understand how cellular systems are integrated into the organism for the purpose of carrying out some physiological function. For instance, an agonist to a cellular receptor induces the cell to make more of an essential product such as a hormone. Sometimes multiple cells cooperate to achieve a goal as in the innate and adaptive immune system.

Here, we introduce a new paradigm wherein enzymes that are part of a metabolic cascade also serve as receptors on stem cells. Importantly, the agonists for these enzymes are their otherwise metabolic substrates. When stem cells bearing these enzymes on their surface are agonized, they differentiate into cells with properties that synergize with the “mission” of the metabolic cells that also use them, albeit as soluble enzymes. It seems that the physiological logic is compelling. There are fundamentally three ways to increase the output of a physiological goal. One can increase the output of a given set of cells, make more of these same cells, or induce a new set of cells of which the “mission” complements that of the other cells. This latter case seems to operate for the thyroid as shown here. By using enzymes that are part of the thyroid hormone metabolic pathway as receptors on stem cells, the synergistic cellular systems become beautifully integrated. When more thyroid hormone is produced, that event is accompanied by increased production of substrates for one of the pathway’s metabolic enzymes that also functions as a receptor on stem cells. The net result is that one gets more thyroid hormone as well as new brown adipocyte-like cells, both of which inure to thermogenesis.

Thyroid hormone signaling is strongly related to brown adipocyte differentiation and thermogenic function [14]. Brown adipose tissue is an important thermogenic organ [15], and brown adipocytes have multiple small lipid droplets, or intracellular fat, as opposed to white adipocytes that have a single massive lipid droplet [16]. The brown adipocyte marker, UCP1, plays an important role in the production of heat during adaptive thermogenesis by regulating mitochondrial uncoupling [17] and by increasing mitochondrial number [18,19]. In this study, we found that H3 Ab induces stem cells to differentiate into cells that have the morphology and expression pattern of brown adipocytes, suggesting that H3 Ab is an agonist antibody for brown adipocyte differentiation. In the mouse, these differentiated cells migrate to heart tissue and increased mitochondrial number.

Studies over several decades have argued that functional human brown adipocytes exist and are important in adult humans [20,21,22,23,24,25]. Recent studies have also reported that brown adipocytes play an important role in regulating cardiovascular health [26,27,28]. Therefore, the heart is an important target organ for the thyroid hormone since both increased and decreased hormone action in the heart can lead to alteration of heart rate, ventricular contractility, cardiac output, and vasodilation [17]. If IYD performs its deiodination reaction at the cell membrane in cardiovascular tissue, it may facilitate effective homeostatic regulation of thyroid hormone function. Since heart muscle cells depend heavily on mitochondrial metabolism to provide energy needed for cardiac function, IYD expression and the agonist action of H3 Ab to enrich mitochondria in heart tissue through differentiation of HSCs into brown adipocytes may be of therapeutic benefit. Our results showed that the target antigen IYD was detected by H3 Ab in human myocardium, especially around the blood vessels where the brown adipocyte marker UCP1 is also heavily expressed.

Reconstituting human tissues, generating new desired cells, and directing cells to specific tissues and integrating them into sites of injury are vitally important for regenerative medicine. Previous studies have shown that mouse, zebrafish, and human cardiomyocytes have the capacity to regenerate and migrate to injured hearts using growth factors, cytokines, paracrine, exosomes, and stem cells [29,30,31,32,33,34,35]. Since our knowledge about regeneration and migration is still limited, the therapeutic value of H3 Ab to modulate cardiac performance or regeneration is not known.

There are several aspects of this study that need further study. First, it will be important to know if IYD substrates can generate brown adipocytes and can migrate to the heart in an in vivo model. Secondly, we need to understand why the newly induced brown adipocyte-like cells selectively migrate to the heart. Finally, from a quantitative point of view, it will be important to know the magnitude of the stem cell’s contribution to overall thermogenesis or heart disease.

## Supplementary Materials

The supplementary materials are available online at https://www.mdpi.com/2073-4409/9/1/256/s1, Figure S1: Phylogenetic tree generated by DNA sequencing analysis of antibody genes in different organs, Figure S2: An agonist antibody induces cell migration, Figure S3: An agonist antibody regulates cell migration. Figure, S4. An agonist antibody induces more mitochondria in the heart tissues. Table S1: Unique brown and general adipocyte expressed genes, Table S2: Real time PCR primer sequences.
