# Supplementary material for: Agonist Antibody Converts Stem Cells into Migrating Brown Adipocyte-Like Cells in Heart
Source: Cells. 2020 Jan 20;9(1):256. doi: 10.3390/cells9010256 (PMC7017361; doi:10.3390/cells9010256)
Supplement: Supplementary file 1 [file cells-09-00256-s001.pdf]

cells

MDPI

Supplementary Materials

Agonist Antibody Converts Stem Cells into Migrating Brown Adipocyte-like Cells in Heart

Kyung Ho Han [1,2], Britni M. Arlian [3], Chih-Wei Lin [1], Hyun Yong Jin [4], Geun-Hyung Kang [5], Sahmin Lee [5], Peter Chang-Whan Lee [2,*] and Richard A. Lerner [1,*]

1 Department of Chemistry, The Scripps Research Institute, La Jolla, CA 92037, USA; kyungho1.han@gmail.com (K.H.H.); cwlin@scripps.edu (C.-W.L.)
2 Department of Biomedical Sciences, University of Ulsan College of Medicine, ASAN Medical Center, Seoul 05505, Korea
3 Departments of Molecular Medicine, Immunology and Microbiology, The Scripps Research Institute, La Jolla, CA 92037, USA; britni@scripps.edu
4 Department of Urology, University of California, San Francisco, CA 94158, USA; jinhyunyong@gmail.com
5 Division of Cardiology, Asan Medical Center Heart Institute, University of Ulsan College of Medicine, Seoul 05505, Korea; rkdrmsgud@nate.com (G.-H.K.); sahmin.lee@amc.seoul.kr (S.L.)
* Correspondence: pclee@amc.seoul.kr (P.C.-W.L.); rlerner@scripps.edu (R.A.L.); Tel.: +82-2-3010-2799 (P.C.-W.L.); +1-858-784-8265 (R.A.L.)

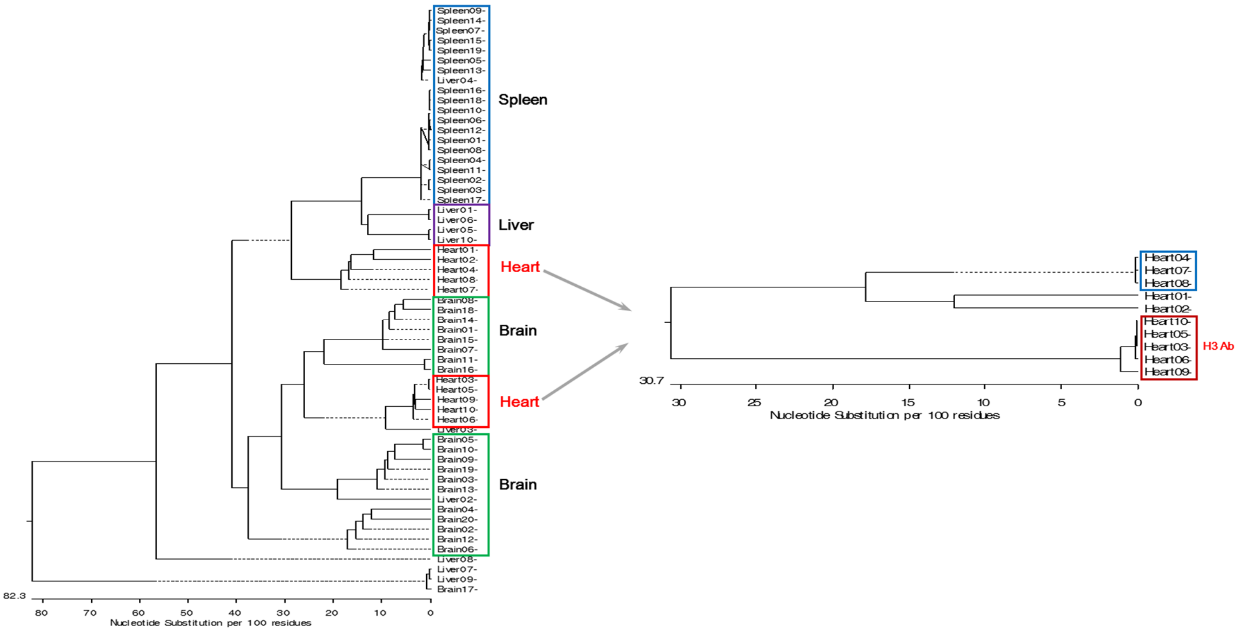

Figure S1. Phylogenetic tree generated by DNA sequencing analysis of antibody genes in different organs. A total of 60 antibody genes were amplified by PCR and sequenced from the brain, heart, liver, and spleen. 10 heart antibody genes could be grouped into 4 major homologs. The H3 gene was most abundant as it appeared 5 times in the heart.

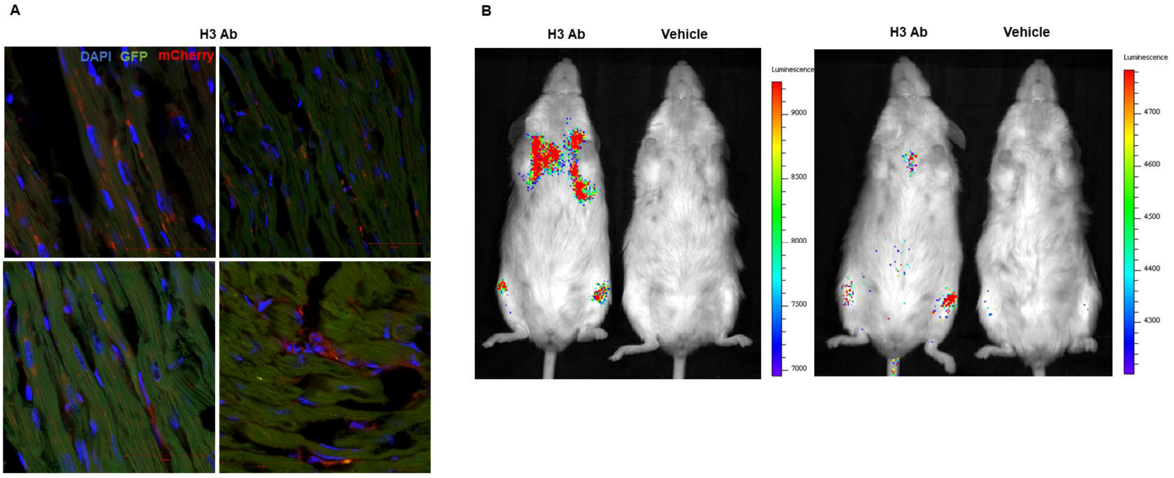

Figure S2. An agonist antibody induces cell migration. (A) Bone marrow from mCherry⁺ mice was adoptively transplanted into lethally irradiated GFP⁺ mice, which were then injected with H3 Ab (50 µg/mouse, i.p., two times/week) for 3 weeks. Following the treatment, hearts were harvested from perfused mice and analyzed by immuno-fluorescence histochemistry. GFP⁺ heart tissue sections (10 µm) were incubated with anti-mCherry and DAPI antibodies and then scanned by confocal microscopy. Scale bars = 50 µm. (B) Representative images (1 week post-transplantation) of FVB/NJ mice transplanted with luc⁺ bone marrow cells that were infected *in vitro* with the H3 Ab lentiviral vector or no Ab (control).

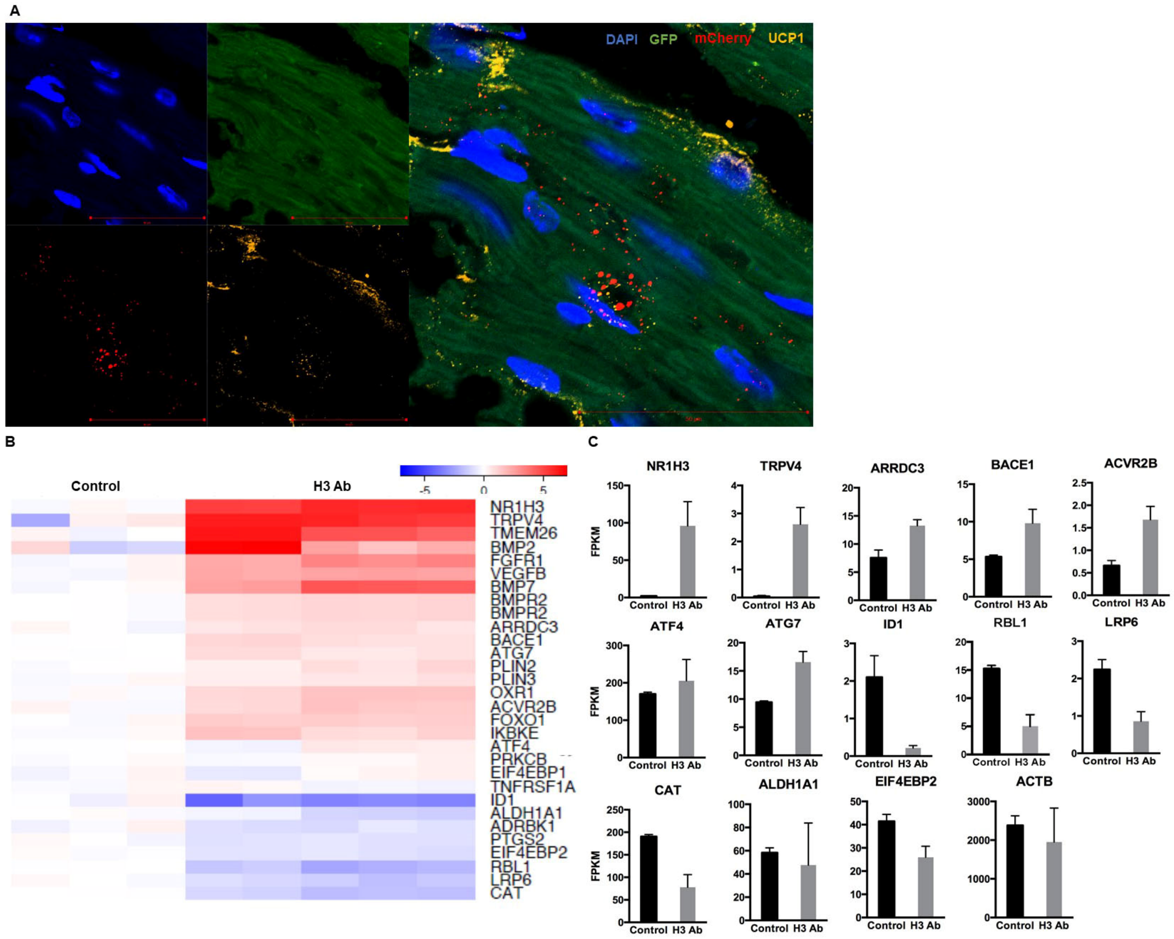

Figure S3. An agonist antibody regulates cell migration. (A) Bone marrow from mCherry$^+$ mice was adoptively transplanted into lethally irradiated GFP$^+$ mice, which were then injected with H3 Ab (50 µg/mouse, i.p., two times/week) for 3 weeks. Following the treatment, hearts were harvested from perfused mice by 2% PFA and analyzed by immuno-fluorescence histochemistry. GFP$^+$ heart tissue sections (10 µm) were incubated with anti-mCherry, DAPI, and anti-UCP1 antibodies and then scanned by confocal microscopy, suggesting that brown adipocyte migrated from the bone marrow to the heart. mCherry$^+$ cells co-stained for the UCP1 brown adipocyte marker, suggesting the mCherry$^+$ donor cells that migrated from the bone marrow to the heart were brown adipocyte-like cells. Scale bars = 50 µm. (B) Brown adipocyte gene expression as demonstrated by RNA sequencing assay. The hierarchal heat map clustering shown that the brown adipocyte have a distinctive pattern versus vehicle Ab treated human CD34+ cells. The expression profile of the induced brown adipocytes is consistent with previous reports. (C) Highly expressed brown adipocyte markers from the RNAseq analysis (n = 3 and 5) are summarized as Fragments Per Kilobase of transcript per Million mapped reads (FPKM).

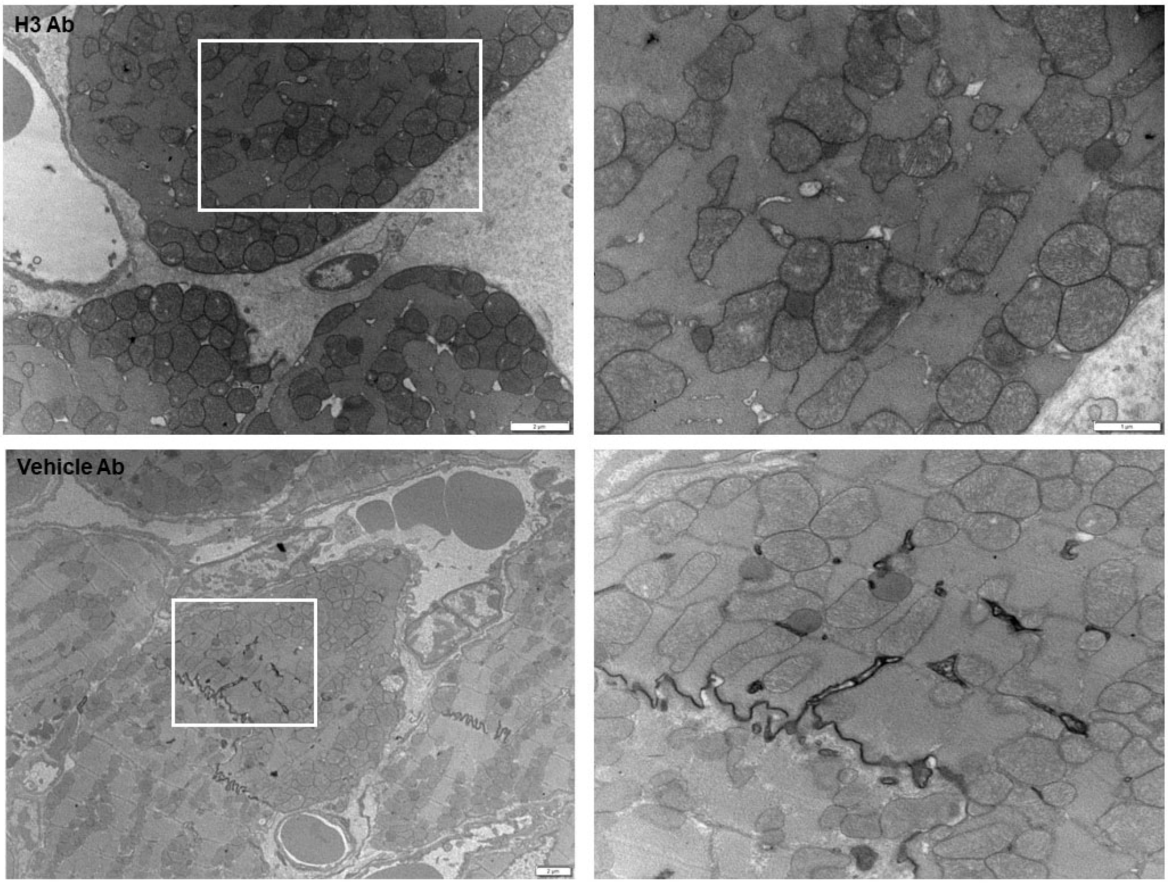

Figure S4. An agonist antibody induces more mitochondria in the heart tissues. (A) After H3 Ab (50 µg/mouse i.p. two times/week) was transplanted C57BL6 mice for 3 weeks, the hearts were scanned by electron microscopy. The white boxes indicate the images correspond to the adjacent magnified images. Scale bars = 1 or 2 µm.

Table S1. Unique brown and general adipocyte expressed genes.

Gene	Control-1	Control-2	Control-3	H3Ab-1	H3Ab-2	H3Ab-3	H3Ab-4	H3Ab-5	Class
BMP7	0.1	0.1	0.1	0.6	0.6	2.4	2.2	2.0	Brown
CEBPB	17.1	35.2	18.8	100.3	94.9	160.9	238.8	239.4	Brown
PDK4	1.4	1.0	1.2	16.7	22.6	9.2	10.5	15.1	Brown
EPSTI1	1.9	2.0	2.3	14.7	13.9	7.9	9.0	5.5	Brown
PPARA	1.7	1.8	1.6	4.7	4.0	7.0	6.7	6.5	Brown
SHOX2	0.1	0.1	0.1	6.3	6.4	15.6	15.9	20.1	General
TMEM26	0.2	0.1	0.1	11.4	11.0	3.6	3.5	2.5	General
FABP4	0.1	0.5	0.3	259.8	306.2	6.1	4.1	8.9	General
PPARG	2.6	4.7	3.5	10.7	10.5	15.0	14.4	17.1	General
NR1H3	1.9	2.0	2.5	83.8	80.3	130.2	115.0	121.8	Adipocyte
TRPV4	0.0	0.1	0.1	3.3	3.3	2.9	2.2	1.9	Adipocyte
TMEM26	0.2	0.1	0.1	11.4	11.0	3.6	3.5	2.5	Adipocyte
BMP2	0.1	0.0	0.0	3.3	3.7	0.2	0.1	0.1	Adipocyte
FGFR1	1.3	1.8	1.4	7.4	6.9	15.1	12.5	15.9	Adipocyte
VEGFB	7.5	10.5	7.0	44.1	40.1	46.8	51.5	46.3	Adipocyte
BMP7	0.1	0.1	0.1	0.6	0.6	2.4	2.2	2.0	Adipocyte
BMPR2	5.8	5.4	6.0	10.5	11.1	12.5	12.3	12.8	Adipocyte
ARRDC3	8.9	6.1	7.7	11.9	13.1	14.4	14.6	12.5	Adipocyte
BACE1	5.1	5.3	5.6	11.2	12.2	9.9	9.2	9.4	Adipocyte
ATG7	9.6	9.5	9.2	18.9	18.0	14.4	14.3	16.5	Adipocyte
PLIN2	50.1	55.5	54.6	70.6	70.9	98.9	86.9	116.0	Adipocyte
PLIN3	35.4	48.1	38.2	55.9	55.4	63.2	63.3	72.0	Adipocyte
OXR1	17.2	16.8	20.4	37.0	38.9	56.8	57.6	55.2	Adipocyte
ACVR2B	0.8	0.6	0.6	1.3	1.4	2.1	1.8	1.7	Adipocyte
FOXO1	1.3	1.6	1.2	3.6	3.4	3.7	3.3	3.5	Adipocyte
IKBKE	5.6	6.8	5.8	19.6	18.3	11.6	11.1	14.0	Adipocyte
ATF4	167.2	175.8	167.2	140.7	133.0	268.2	250.9	234.8	Adipocyte
PRKCB	22.9	27.8	23.7	22.1	22.6	28.4	28.4	32.7	Adipocyte
EIF4EBP1	29.1	45.7	34.3	24.5	23.8	39.3	44.7	49.4	Adipocyte
TNFRSF1A	54.7	70.0	51.7	71.4	66.5	47.0	49.7	45.1	Adipocyte
ID1	2.1	2.7	1.5	0.1	0.3	0.2	0.2	0.2	Adipocyte
ALDH1A1	59.7	53.7	61.6	48.2	45.6	26.9	24.8	22.4	Adipocyte
ADRBK1	71.6	120.8	87.6	54.4	48.4	45.7	60.2	52.3	Adipocyte
PTGS2	3.5	2.5	3.2	1.7	1.8	1.9	1.8	1.8	Adipocyte
EIF4EBP2	44.8	40.3	39.2	24.3	24.5	26.1	23.7	21.8	Adipocyte
RBL1	14.8	15.0	15.9	5.2	5.9	2.8	3.3	4.0	Adipocyte
LRP6	2.5	2.0	2.2	1.2	1.1	0.9	0.7	0.8	Adipocyte
CAT	192.0	185.4	193.9	89.7	82.1	53.4	58.8	57.7	Adipocyte
ACTB	2228.1	2667.5	2247.0	2450.8	2382.0	1091.1	1149.8	1383.0	house keeping

FPK values for unique brown and general adipocyte markers.

Table S2. Real time PCR primer sequences.

Gene name	Forward	Reverse	Species
UCP1	ACTGCCACACCTCCAGTCATT	CTTTGCCTCACTCAGGATTGG	human
PRDM16	CAGCACGGTGAAGCCATTC	GCGTGCATCCGCTTGTG	human
PGC1α	CCCTGCCATTGTTAAGACC	TGCTGCTGTTCCTGTTTTC	human
AP2 (FABP4)	ACAAGCTGGTGGTGGAATGTG	CCTTTGGCTCATGCCCTTT	human
AdipoQ	CGATTGTCAGTGGATCTGACG	CAACAGTAGCATCCTGAGCCCT	human
PPAR-γ	GTGCCAGTTTCGATCCGTAGA	GGCCAGCATCGTGTAGATGA	human
18s rRNA	GGCCCTGTAATTGGAATGAGTC	CCAAGATCCAACTACGAGCTT	human
